# Sex‐specific accelerated decay in time/activity‐dependent plasticity and associative memory in an animal model of Alzheimer's disease

**DOI:** 10.1111/acel.13502

**Published:** 2021-11-18

**Authors:** Sheeja Navakkode, Jessica Ruth Gaunt, Maria Vazquez Pavon, Vibhavari Aysha Bansal, Riya Prasad Abraham, Yee Song Chong, Toh Hean Ch'ng, Sreedharan Sajikumar

**Affiliations:** ^1^ Lee Kong Chian School of Medicine Nanyang Technological University Singapore Singapore; ^2^ Department of Physiology National University of Singapore Singapore Singapore; ^3^ School of Biological Science Nanyang Technological University Singapore Singapore; ^4^ Healthy Longevity Translational Research Programme Yong Loo Lin School of Medicine National University of Singapore Singapore Singapore; ^5^ Life Sciences Institute Neurobiology Programme National University of Singapore Singapore Singapore

**Keywords:** Alzheimer's disease, behavioural tagging, LTP, sexual dimorphism, STDP, synaptic plasticity, synaptic tagging and capture, transcriptome profiling

## Abstract

Clinical studies have shown that female brains are more predisposed to neurodegenerative diseases such as Alzheimer's disease (AD), but the cellular and molecular mechanisms behind this disparity remain unknown. In several mouse models of AD, synaptic plasticity dysfunction is an early event and appears before significant accumulation of amyloid plaques and neuronal degeneration. However, it is unclear whether sexual dimorphism at the synaptic level contributes to the higher risk and prevalence of AD in females. Our studies on APP/PS1 (*APPSwe*/*PS1dE9*) mouse model show that AD impacts hippocampal long‐term plasticity in a sex‐specific manner. Long‐term potentiation (LTP) induced by strong tetanic stimulation (STET), theta burst stimulation (TBS) and population spike timing‐dependent plasticity (pSTDP) show a faster decay in AD females compared with age‐matched AD males. In addition, behavioural tagging (BT), a model of associative memory, is specifically impaired in AD females with a faster decay in memory compared with males. Together with the plasticity and behavioural data, we also observed an upregulation of neuroinflammatory markers, along with downregulation of transcripts that regulate cellular processes associated with synaptic plasticity and memory in females. Immunohistochemistry of AD brains confirms that female APP/PS1 mice carry a higher amyloid plaque burden and have enhanced microglial activation compared with male APP/PS1 mice. Their presence in the diseased mice also suggests a link between the impairment of LTP and the upregulation of the inflammatory response. Overall, our data show that synaptic plasticity and associative memory impairments are more prominent in females and this might account for the faster progression of AD in females.

## Graphical abstract

Sex‐specific study of synaptic plasticity and memory reveals female APP/PS1 mice carry a higher amyloid plaque burden and enhanced Iba‐1‐positive microglial activation compared with male APP/PS1. In addition, synaptic plasticity and associative memory impairments are also more prominent in females than in males.

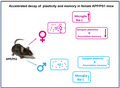

**Abbreviations**
AD: Alzheimer’s disease
LTP: Long‐term potentiation
STET: Strong tetanic
TBS: Theta burst Stimulation
BT: Behavioural tagging
pSTDP: population spike timing‐dependent plasticity
Aβ: Amyloid‐beta
NFTs: neurofibrillary tangles
GWAS: Genome‐wide association studies
aCSF: artificial cerebrospinal fluid
fEPSP: field excitatory postsynaptic potentials
OF: open field
IA: Inhibitory Avoidance
DEGs: differentially expressed genes
FDR: false discovery rate
GO: gene ontology
PRPs: plasticity‐related proteins
LFCs: log2 fold changes

## 1 INTRODUCTION

Alzheimer's disease (AD) is a progressive neurodegenerative disorder characterized by memory loss and behavioural deficits (Latimer et al., 2021; Nardini et al., 2021). The hallmarks of AD include neurodegeneration, the presence of extracellular amyloid‐beta (Aβ) proteins and intracellular neurofibrillary tangles (NFTs) made up of abnormally phosphorylated tau protein (Islam Khan et al., 2018; Mueed et al., 2019; Silva et al., 2019). Although much progress has been made on the molecular basis of AD in the past few decades, it is still not clear how the confluence of sex differences and other risk factors influences the progression of the disease. It is well known that AD differentially affects males and females (Dennison et al.,^2021^). The risk of AD in females is 1 in 6, while in men, it is 1 in 11 (Regitz‐Zagrosek & Seeland, 2012). The factors that might contribute to sex differences in AD are diverse and include differences in genetic background, hormone secretion, activation of microglia and the neuroinflammatory response during disease progression (Loeffler, 2021; Mielke, 2018; Watzka et al., 1999).

Genetic variation plays a major role in the relationship between sex and AD pathology (Breijyeh & Karaman, 2020). Healthy individuals with a maternal AD history are known to show more prominent phenotypic changes in vulnerable brain regions compared to those with a paternal AD history (Berti et al., 2011; Mosconi et al., 2010). The ε4 allele of the apolipoprotein E gene is one of the most common genetic risk factors for AD (Corder et al., 2004). Genetic variants of the apolipoprotein E gene have been shown to confer different risks for male and female AD patients (Corder et al., 2004; Johnson et al., 1998; Payami et al., 1996).

Other major contributing factors to sex differences in the progression of AD are the differences in microglial activation and elevation of the neuroinflammatory response (Manji et al., 2019). Genome‐wide association studies (GWAS) have shown that mutations in genes associated with neuroinflammation are a major risk factor for AD (Zhang et al., 2013). Many of these AD risk factors converge on the microglia, which suggests that microglial activation could be a causal factor for AD (Frigerio et al., 2019). Age‐related expression of hippocampal neuroinflammatory genes is also known to be sexually dimorphic (Mangold et al., 2017).

Along with genetic and microglial influences, structural differences at the synapse can also potentially contribute to sex‐specific risks of neurodegenerative diseases (Zheng et al., 2019). Reports have indicated that the CA3 pyramidal cells of males and females are distinct in structure, function and plasticity (Scharfman & MacLusky, 2017). Moreover, studies have shown that memory‐related synaptic plasticity is sexually dimorphic and this is reflected in the CA1‐dependent spatial behaviour and predisposition to neuropsychiatric disorders such as AD (Wang et al., 2018). Induction of long‐term potentiation (LTP), a cellular correlate of learning and memory in the CA1 area of hippocampus, was shown to be influenced by the interaction of gonadal hormones (Yang et al., 2004), and it has been demonstrated that, in female, but not male rodents, LTP and its associated kinases require endogenous expression of oestrogen and its receptor, to determine the threshold for the induction of LTP and spatial memory (Yang et al., 2004). There is evidence that synaptic plasticity is modulated by a number of sex‐specific signalling mechanisms that vary depending on the brain region (Hyer et al., 2018). The effects of sexual dimorphism on synaptic plasticity and memory and its contribution to the higher incidence of AD in females are not well understood. For example, we still do not fully understand the relationship between sex‐specific plasticity mechanisms and changes in the brain microenvironment during AD progression, and whether the interaction between these two processes contributes towards the accelerated AD pathology observed in females.

To understand how synaptic plasticity is involved in the increased vulnerability of females to AD, we examined whether AD affects the induction and maintenance of different forms of long‐lasting LTP in males and females. We specifically examined protein synthesis‐dependent late LTP (L‐LTP) induced by strong tetanus (STET), theta burst stimulation (TBS) and population spike timing‐dependent plasticity (pSTDP) in both female and male WT and APP/PS1 mice. We used 4‐ to 5‐month‐old mice for our studies, as this is a time point at which synaptic deficits start to appear but well before widespread neuronal death (Sadowski et al., 2004; Sun et al., 2019). Behavioural tagging (BT) paradigm was used to test associative memory in both sexes from WT and APP/PS1 mice. Our results show that overall, APP/PS1 mice have impaired L‐LTP and associative memory, with female APP/PS1 mice showing a faster decay in plasticity and memory compared with male mice. Transcriptome profiling of male and female hippocampus indicated that AD mice, particularly female mice, have a robust upregulation of immune‐related genes and microglial activation, which was confirmed with immunohistochemistry. In addition, we observed decreased expression of genes associated with neuronal plasticity exclusively in female mice. Collectively, our data show that synaptic plasticity impairment is more pronounced in females than in males, and this likely contributes to greater cognitive impairments and increased vulnerability to AD.

## 2 MATERIALS AND METHODS

### 2.1 Electrophysiology

#### 2.1.1 Animals

All animal procedures were approved by the Institutional Animal Care and Use Committee (IACUC) of the National University of Singapore. We used a mouse model of AD that expresses a mutated chimeric mouse/human APP and the exon‐9‐deleted variant of human PS1, both linked to familial AD, under the control of a prion promoter element (*APPSwe*/*PS1dE9*), which we denote as APP/PS1 (Borchelt et al., 1997). We used 4‐ to 5‐month‐old animals as it is an early stage when synaptic plasticity and behavioural changes occur along with Aβ pathology (Gong et al., 2004; Yu et al., 2019). We did not use younger animals as there are mixed reports on the plasticity deficits at 2–3 age group depending on the mouse model used (Corder et al., 2004; Trinchese et al., 2004). 70 animals were used to isolate 168 slices for electrophysiology experiments in this study, out of which 33 were from WT males, 38 from WT females, 46 from APP/PS1 males and 51 from APP/PS1 females. For behavioural experiments, 56 animals were used with 14 animals in each group. For immunohistochemistry and transcriptome analysis, 32 animals were used, with eight animals from each group. Thus, a total number of 158 animals were used for this study. Animals were housed under 12‐h light/12‐h dark conditions with food and water available ad libitum.

#### 2.1.2 Hippocampal slice preparation

Animals were anaesthetized briefly using CO_2_ and were decapitated, and their brains were quickly removed and transferred to 4°C artificial cerebrospinal fluid (aCSF)‐a modified Krebs–Ringer solution containing the following (in mM): 124 NaCl, 3.7 KCl, 1.2 KH_2_PO_4_, 1 MgSO_4_·7H_2_O, 2.5 CaCl_2_·2H_2_O, 24.6 NaHCO_3_ and 10 d‐glucose. The pH of aCSF was between 7.3 and 7.4 when bubbled with 95% oxygen and 5% carbon dioxide (carbogen). Both right and left hippocampi were isolated out in the cold (2–4°C) aCSF being continuously bubbled with carbogen (Krishna‐K et al., 2020; Shetty et al., 2015). Transverse hippocampal slices of 400 μm thickness were prepared from the right and left hippocampi using a manual tissue chopper (Stoelting), and transferred onto a nylon net in an interface chamber (Scientific Systems Design) and incubated at 32°C with an aCSF flow rate of 1 ml/min and carbogen consumption of 16 L/h. The entire process of animal dissection, hippocampal slice preparation and placement of slices on the chamber was done within approximately 5 min to ensure that hippocampal slices were in good condition for electrophysiology studies. The slices were incubated for at least 3 h before starting the experiments (for more details, see Sajikumar et al., 2005; Shetty et al., 2015).

#### 2.1.3 Field potential recordings

**FIGURE 1:**
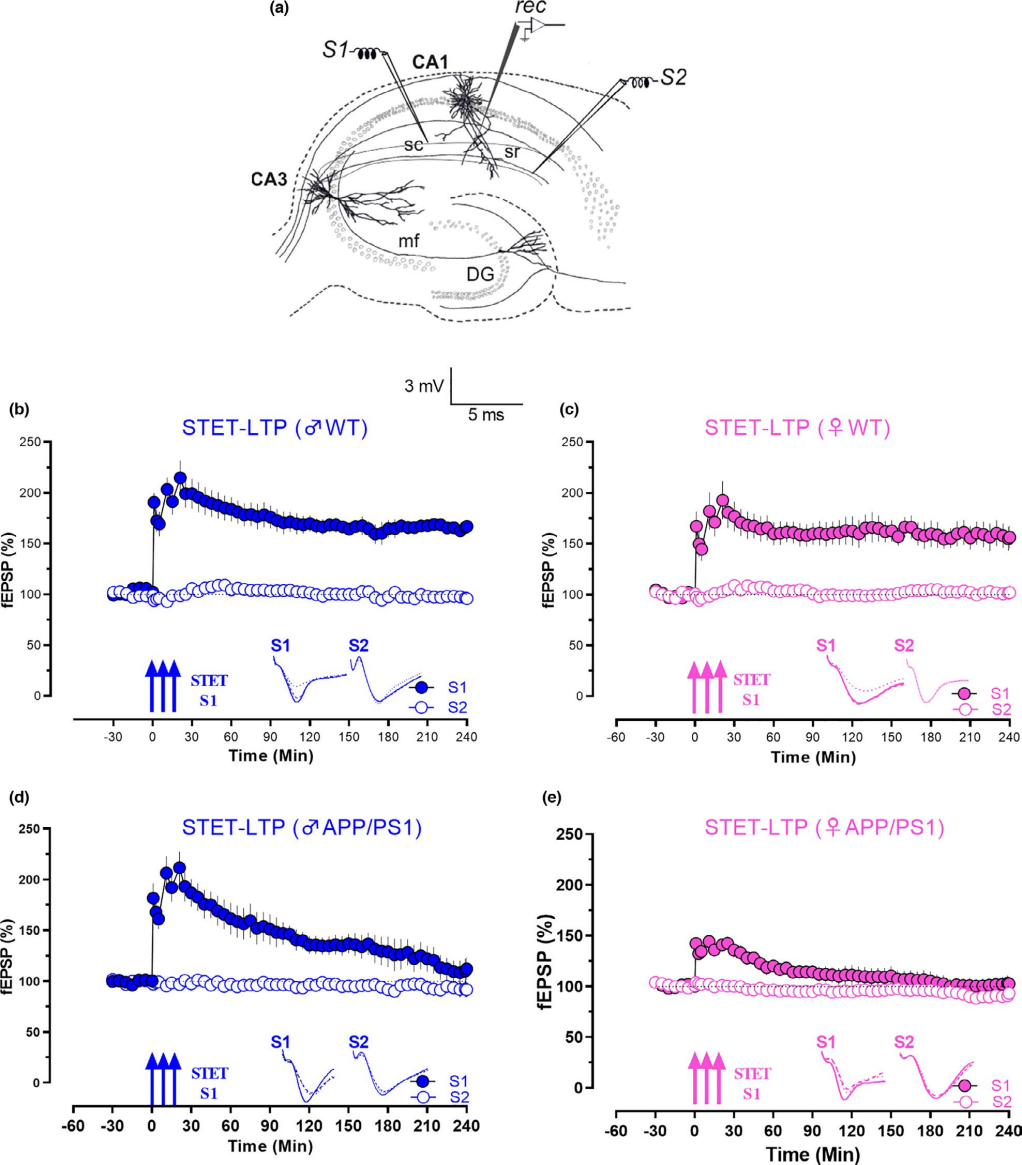
APP/PS1 females show a faster decay in L‐LTP induced by STET (a) Schematic representation of the location of electrodes in the CA1 region of a transverse hippocampal slice. Recording electrode (rec) positioned in CA1 apical dendrites was flanked by two stimulating electrodes S1 and S2 placed in the stratum radiatum (sr) layer to stimulate two independent Schaffer collateral (sc) synaptic inputs of the same neuronal population. (b) The STET in S1 (blue, filled circles) resulted in a significant potentiation that maintained for 4 h, while the control potentials in S2 (blue, open circles) remained stable throughout the recording in male WT mice (n = 6). (c) STET in S1 (pink filled circles) resulted in a long‐lasting LTP for 4 h, while the control input S2 (pink, open circles) was stable for the time course investigated in female WT mice (n = 7). (d) STET in S1 (blue, filled circles) only resulted in E‐LTP that gradually decayed to baseline in male APP/PS1 mice, (180‐min Wilcox, *p* = 0.07, U test, *p* = 0.08; blue, filled circles; n = 7). (e) In female APP/PS1 mice, STET in S1 also resulted in an early form of LTP, which decayed to baseline (95‐min Wilcox, *p* = 0.109, U test, *p* = 0.07; n = 7). Control stimulation of S2 in both d and e showed stable potentials for the recorded time period (open circles). Error bars in all the graphs indicate ±SEM. Analogue traces represent typical fEPSPs of inputs S1 and S2, recorded 15 min before (dotted line), 30 min after (dashed line) and 240 min (solid line) after tetanization. Three solid arrows represent the time of induction of L‐LTP by STET for the induction of late LTP. Scale bars: vertical, 2 mV; horizontal, 3 ms

In all the electrophysiology recordings, two‐pathway experiments were performed. Two monopolar lacquer‐coated stainless steel electrodes (5 MΩ; AM Systems) were positioned at an adequate distance within the stratum radiatum of the CA1 region for stimulating two independent synaptic inputs S1 and S2 of one neuronal population, thus evoking field excitatory postsynaptic potentials (fEPSP) from Schaffer collateral/commissural‐CA1 synapses (Figure 1a). Pathway specificity was tested using the method described in Sajikumar and Korte (2011). A third electrode (5 MΩ; AM Systems) was placed in the CA1 apical dendritic layer for recording fEPSP. After the pre‐incubation period, a synaptic input–output curve (afferent stimulation vs. fEPSP slope) was generated. Test stimulation intensity was adjusted to elicit fEPSP slope of 40% of the maximal slope response for both synaptic inputs S1 and S2. The signals were amplified by a differential amplifier, digitized using a CED 1401 analogue‐to‐digital converter (Cambridge Electronic Design) and monitored online with custom‐made software. To induce late LTP, a “strong” tetanization (STET) protocol consisting of three trains of 100 pulses at 100 Hz (single burst, stimulus duration of 0.2 ms per polarity), with an intertrain interval of 10 min, was used. Theta burst stimulation–LTP (TBS‐LTP) was induced using a protocol, which consisted of 50 bursts (consisting of four stimuli) at an interstimulus interval of 10 ms. The 50 bursts were applied over a period of 20 s at 5 Hz.

In all experiments, a stable baseline was recorded for at least 30 min using four 0.2‐Hz biphasic constant current pulses (0.1 ms per polarity) at each time point. Four 0.2‐Hz biphasic, constant current pulses (spaced at 5 s) given every 5 min were used for postinduction recordings, and the average slope values from the four sweeps were considered as one repeat and used for plotting fEPSP percentage vs time graphs. Initial slopes of fEPSPs were expressed as percentages of baseline averages.

### 2.2 Population spike timing‐dependent plasticity

**FIGURE 2:**
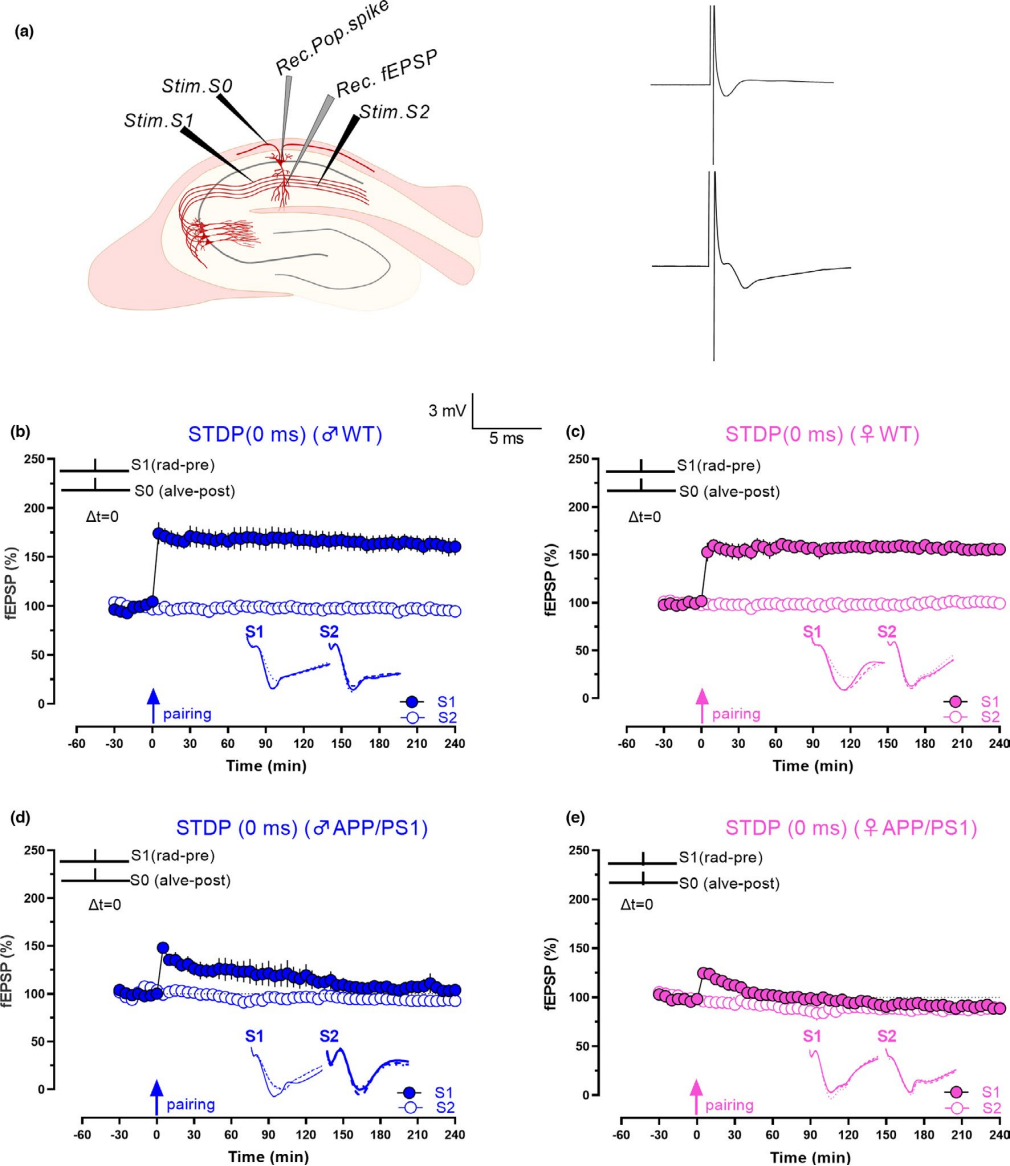
Faster impairment of pSTDP in female APP/PS1 mice with coincidental pre‐ and postsynaptic stimulation. (a) Schematic diagram of location of electrodes in a hippocampal slice for STDP experiments. The two grey inverted triangles represent recording electrodes placed in the cell body and dendritic layer of CA1 area hippocampal slices to record population spike and fEPSP, respectively. Black inverted triangles represent three stimulating electrodes. Stimulating electrodes 1 and 2 (Stim. S1 and Stim. S2) were placed in the stratum radiatum to stimulate two independent SC pathways, and Stim. S0 represents a stimulating electrode located in the alveus layer to evoke antidromic action potentials in CA1 pyramidal neurons. Analogue traces on the right side represents field potentials that were recorded from the stratum pyramidale (upper trace) and stratum radiatum (lower trace) in response to coincidental pairing of alveus (Stim. S0) and stratum radiatum (Stim. S1) stimulation. (b) Simultaneous stimulation of the presynaptic input S1 and the alveus layer (S0; 20 pairings, 1 Hz) induced a persistent pathway‐specific increase in synaptic responses of input S1 (blue, filled circles) that lasted 4 h. Unpaired input S2 (blue, open circles) remained stable in male WT mice (n = 6). (c) Simultaneous stimulation in female WT slices also resulted in long‐lasting increase in synaptic responses that lasted for 4 h in paired input S1 (pink, filled circles), while the unpaired control input remained stable (pink, open circles; n = 10). (d) Pairing at 0 ms in male APP/PS1 mice showed only a decremental LTP (70‐min Wilcox, *p* = 0.02, U test, *p* = 0.1; n = 6). (e) Pairing at 0 ms in female APP/PS1 mice also resulted only in a decremental LTP (40‐min Wilcox, *p* = 0.2, U test, *p* = 0.08; n = 8). In all experiments, the control unpaired input was stable throughout the time period of investigation (open blue and pink circles). Relative timing Δt represents the time points at which stimulus S0 (postsynaptic component) and S1 (presynaptic component) were initiated. Solid single arrow represents the time of pairing. Scale bars: vertical, 2 mV; horizontal, 3 ms. Error bars indicate ±SEM. Symbols and analogue traces as in Figure 1

For population spike timing‐dependent plasticity (pSTDP) experiments, a stimulating electrode (S0) was positioned in the alveus to evoke antidromic neuronal action potentials, similar to our earlier report (Pang et al., 2019). The antidromic spikes induced by the stimulation of the alveus lead to backpropagating action potentials within the dendrites of CA1 pyramidal neurons. The alveus stimulation regulates the degree of postsynaptic neuronal activity. In addition to the alveus stimulating electrode, two stimulating electrodes, S1 and S2, were located in the stratum radiatum of the CA1 region to stimulate Schaffer collateral/commissural fibres. Two recording electrodes were positioned in the CA1 stratum pyramidale and stratum radiatum to record population spikes and field excitatory postsynaptic potentials (fEPSPs) from the Schaffer collateral/commissural‐CA1 synapses, respectively (Figure 2a). The test stimulation strength at S1 was set at a subthreshold intensity for population spike generation. An input–output curve was determined for synaptic input S2 (afferent stimulation versus population spike amplitude or fEPSP). Pathway specificity of two inputs, S1 and S2, was always determined by paired‐pulse stimulations described in Krishna‐K et al., 2020; Pang et al., 2019. At S2, the test stimulation strength was set to produce a population spike amplitude of 40% of the maximal response. For the stimulus in S0, the intensity of stimulation was set to elicit an antidromic response of approximately 2 mV in the CA1 pyramidal neurons.

20 pairs of single stimuli (stimulus duration of 0.2 ms/ polarity) at 1 Hz were delivered to S0 and S1 at distinct relative time intervals for different sets of experiments (Δt = t0‐t1, where t0 and t1 are the times at which stimuli S0 (postsynaptic component) and S1 (presynaptic component) were initiated (Pang et al., 2019).

### 2.3 Oestrous cycle phase

The oestrous cycle phase of female WT and APP/PS1 mice was identified by using vaginal smears. The vaginal canal opening was first rinsed with distilled water, and a mild penetration of vaginal orifice was done using a pipette and saline (50 µl) was flushed into the vagina gently three to four times with a pipette. The oestrous cycle phase was determined from the vaginal flush using the crystal violet staining as described in McLean et al., 2012. The vaginal smear was allowed to dry at room temperature. Then, the slide was dipped in crystal violet for 1 min and washed in distilled water for 1 min. This was repeated once more. The oestrous cycle was identified by looking at the variable proportion of leukocytes, cornified cells and nucleated epithelial cells. The smears having predominantly leukocytes were classified as dioestrous. Smears having predominantly nucleated epithelial cells and very few or no leukocytes were classified as pro‐oestrous and the smears having mainly cornified cells were classified as oestrous (McLean et al., 2012).

The levels of gonadal hormones during pro‐oestrous cycle is approximately 60 pg/ml, which is about twice that at the other three stages namely oestrus, metoestrus and dioestrus, with oestrogen levels of around 33–38 pg/ml (Chen et al., 2009). We grouped animals into high oestrogen (pro‐oestrus), low oestrogen or non‐pro‐oestrus, which comprises (oestrus, metoestrus and dioestrus).

### 2.4 Behavioural tagging

**FIGURE 3:**
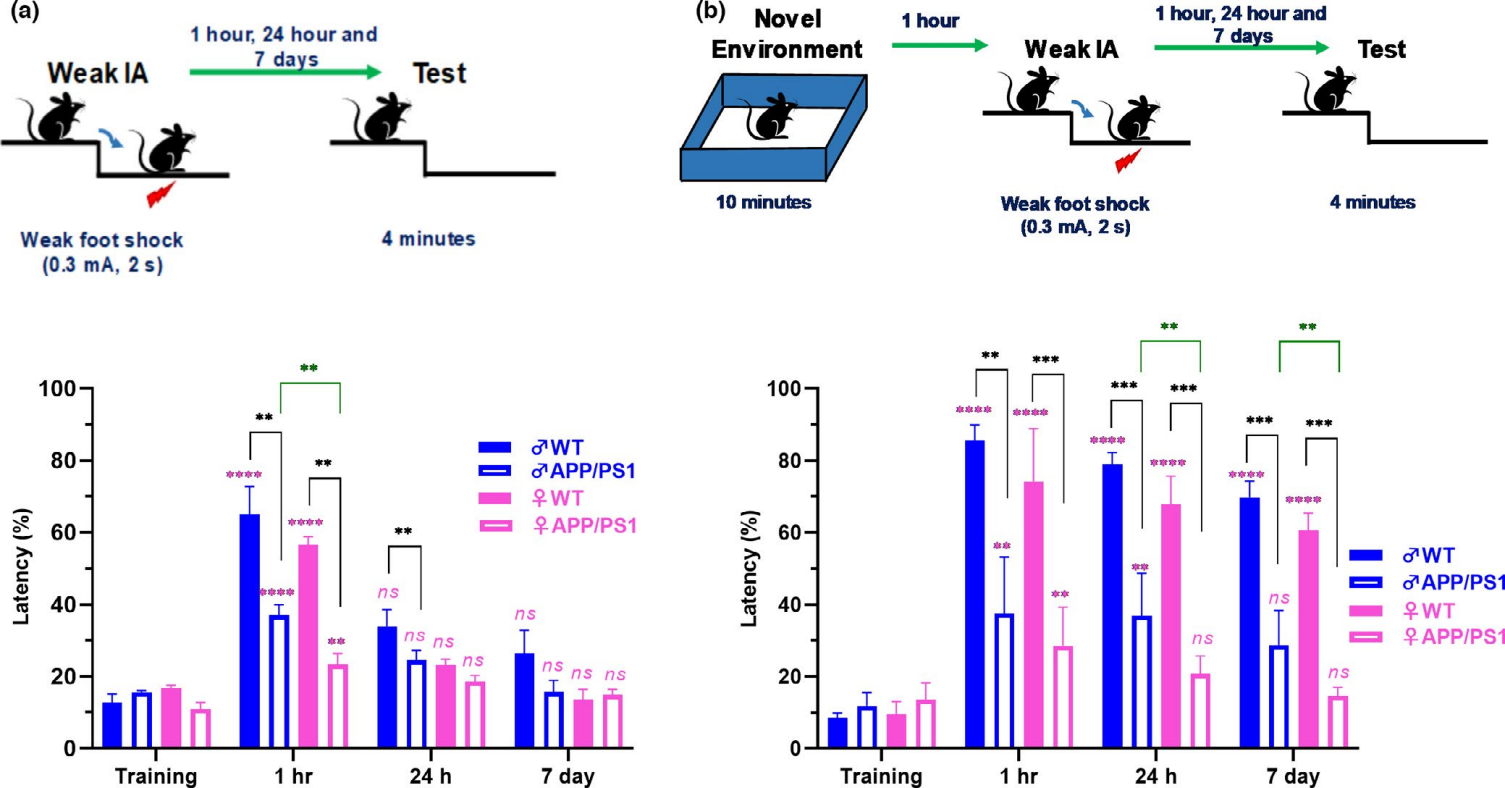
Impairment of associative memory in female APP/PS1 mice. (a) Schematic diagram of the experimental protocol used for control BT paradigm. A mouse was given weak IA training by providing a weak foot shock consisting of 0.3 mA for 2 s. Step‐down latency was tested at 1, 24 h and 7 days post‐IA. Memory for IA learning was observed only at 1 h in all four groups: WT males (filled blue bars); APP/PS1 males (open blue bars); WT females (filled pink bars); and APP/PS1 females (pink open bars; n = 7 from all groups). (b) Schematic diagram of the experimental protocol used for BT paradigm. Mice were given weak IA training, 1 h after exposure to a novel environment (open field, OF) for 10 min. Step‐down latency was tested at 1, 24 h and 7 days post‐IA. Associative memory was observed in both WT males and females (filled blue and pink bars, respectively) and in APP/PS1 males (open blue bars) at 24 h, while in APP/PS1 females (open pink bars), associative memory was impaired (n = 7 from all groups). Statistical analyses for latencies at each time point (1, 24 h and 7 days) for all groups were compared against their training period (asterisks in pink). Further statistical tests were performed between WT and APP/PS1 animals (black asterisks), as well as between male and female APP/PS1 mice (green asterisks). Error bars indicate ±SEM. Asterisks indicate significant differences between groups (ns, not significant, **p < 0.01, ***p < 0.001 and ****p < 0.0001)

In order to study associative memory, we used a behavioural tagging (BT) paradigm (Moncada & Viola, 2007; Wong et al., 2019). Male and female WT and APP/PS1 mice were used for this study. They were habituated to the room, 12 h before experiments. The mice were placed in an open field (OF) for 10 min. 1 h after OF, inhibitory avoidance (IA) training was given to the mice (Figure 3b). The OF consists of a plastic box with dimensions of 35 (width) ×35 (length) ×35 cm (height). A weak IA training induces only short‐term memory, but this can be consolidated to long‐term memory by the novelty exploration experience, consisting of 10 min of OF, that occurs 1 h before IA (Moncada & Viola, 2007; Wong et al., 2019). The IA apparatus consists of a 50 (width) ×25 (height) ×25 cm (length) Plexiglas box with a 5 (height) ×8 (width) ×25 cm (length) platform on the left end of a series of bars, which constitutes the floor of the box. During the training session, mice were placed on the platform that faces the left rear corner of the box. When they stepped down, putting their four paws on the bars, they received a weak foot shock (0.3 mA, 2 s) after which they were removed from the box and returned to their home cage. Memory was evaluated by comparing the step‐down latency in the training session with that in the test sessions. The cut‐off time for step‐down latency was 4 min. A high step‐down latency indicates that the animal has better memory for IA. Step‐down latency is represented as percentage values similar to our previous reports (Krishna‐K et al., 2020; Wong et al., 2019). Memory was tested at three different time points: 1, 24 h and 7 days after training sessions. The same animals were used to retest at different time points as in previous reports (Krishna‐K et al., 2020; Wong et al., 2019). As a control experiment, mice received weak IA training without OF and memory was evaluated similarly as mentioned above (Figure 3a).

### 2.5 RNA extraction and sequencing

Whole right hippocampi were dissected from N = 4 APP/PS1 mice and N = 2 wild‐type mice of each sex at age 4.5 months, snap‐frozen and stored at −80°C. Hippocampi were homogenized in TRIzol Reagent (Invitrogen). Total RNA was then extracted and column‐purified using the RNeasy kit (Qiagen) according to the user manual. Directional mRNA libraries were prepared and sequenced by NovogeneAIT Genomics on the Illumina NovaSeq 6000 platform (45 M 150‐bp paired‐end reads per sample). Prior to sequencing, RNA quality was assessed by electrophoresis and Agilent 2100 Bioanalyser analysis. All input RNA samples had RIN >8.0.

### 2.6 mRNA sequencing and analyses

RNAseq data were first processed using Trimmomatic (Bolger et al., 2014) to trim Illumina adapters and remove low‐quality or short‐length reads (quality score <15 or length <30 bp). Paired‐end reads were then aligned to the Genome Reference Consortium mouse genome assembly GRCm38 using STAR (Dobin et al., 2013) and quantified by HTseq (Anders et al., 2015) on the Gekko high‐performance computing cluster at Nanyang Technological University. Counts were filtered to retain genes with at least one log2‐transformed count per million mapped reads (log‐CPMs) in a minimum of three samples (N = 15,521), and upper‐quartile normalization was applied. Differential expression (DE) analysis was conducted using edgeR (likelihood ratio test with robust dispersion estimation; Robinson et al., 2010). To reduce the impact of unwanted variation, we used RUVseq (Risso et al., 2014) to identify factors of variation in the expression of empirically defined negative control genes (all genes except the top‐ranked 5000 in a first‐pass differential expression analysis) and we incorporated the top two factors into the model for DE analysis. We analysed differential expression between AD and WT conditions for each sex and both combined, as well as the interaction effect defined as [*(AD*.*F* ‐ *WT*.*F)* ‐ *(AD*.*M* ‐ *WT*.*M*)]. We identified DE genes using a false discovery rate (FDR) threshold of 10%. Hierarchical clustering of DEGs between AP and WT mice was conducted on batch‐corrected CPMs using the Pearson distances with the ward. D2 algorithm. To analyse enriched biological functions, we conducted Kolmogorov–Smirnov tests for GO Biological Process terms on genes ranked by FDR from DE tests using the topGO package (Alexa & Rahnenführer, 2020), using the weight01 algorithm to reduce redundancy between terms (Alexa et al., 2006). To determine whether gene sets were predominantly upregulated or downregulated compared with the control groups, we then calculated direction scores for each term (formula: (ΣNup ‐ ΣNdown)/√(ΣNup + ΣNdown), where Nup and Ndown represent the number of genes in each term with LFCs above and below 0 [up‐ and downregulated, respectively]). For genes in the GO term “regulation of synaptic plasticity” (GO:0048167), we also plotted rankings by FDR alongside Gaussian kernel density estimation to compare the probabilities that these genes were upregulated and downregulated in AD mice of each sex. To identify the cell types in which the genes of interest are most highly expressed, we used the transcriptome database of mouse cortical cell types by (Zhang et al., 2014). Analyses were conducted in R (v4.0.2, R Core Team, 2020), and figures were generated using ggplot2 (Wickham, 2016).

### 2.7 Immunohistochemistry

Hippocampal sections were prepared using a vibratome (4°C at 100 µm). Sections that came off the vibratome were immediately fixed with 4% PFA in PBS overnight at 4°C, washed and kept in cryoprotectant (30% ethylene glycol, 30% glycerol and 10% 0.2 M PB in Milli‐Q) at −80°C. Prior to processing, sections were washed 3X in PBS and permeabilized in 0.1% Triton X in PBS before antigen retrieval (10 mM Tris Base, 1 mM EDTA and 10% Triton X in Milli‐Q at 37°C for 15 min). Sections were washed (5X, 5 min) in 0.1% Triton X in 1X PBS and blocked for 1 h (10% goat serum, 0.1% Triton X in PBS) before incubation with primary antibodies (overnight) followed by secondary antibodies (4 h) with washes (3X, 0.1% Triton X in PBS) in between incubations. All sections were mounted on slides using Aqua‐Poly/Mount. Unless otherwise stated, all protocols were performed at room temperature with gentle rocking with an orbital shaker. Antibodies and dyes used include the following: mouse anti‐beta amyloid antibody (MOAB‐2; Abcam at 1:1000 dilution), mouse anti‐NeuN (Merck‐Millipore at 1:1000), rabbit anti‐Iba‐1 (Wako at 1:1000), rat anti‐FcγRIIb (R&D Systems at 1:500), Alexa Fluor‐conjugated secondary antibodies (ABLife Tech. at 1:1000) and Hoechst 33342 (Thermo Fisher at 1:1000).

### 2.8 Image acquisition and analysis

All widefield images were acquired using Zeiss Axio Scan.Z1 with a 10X objective with a Hamamatsu Orca Flash detector. Magnified images were taken using Zeiss LSM800 inverted scanning confocal microscope using either 40X or 63X plan Apochromat 1.4NA oil immersion objectives. For all widefield images, quantification was done using IMARIS x64 9.6.0. The hippocampal region was first defined by drawing a boundary based on organization of the distinct cell body layers guided by Hoechst nuclear dye labelling. An intensity threshold (lower 10% signal) was employed for all antibodies to remove noise. A size‐exclusion filter based on signal intensity was then used to accurately select substructures within each slice and to exclude any staining artefacts (MOAB‐2: 10 µm^2^; Iba‐1: 3 µm^2^; NeuN: 3 µm^2^). The total count per slice, average intensity and area for each substructure were compiled, analysed and graphed as appropriate. For plaque burden and microglia count, the total counts for each slice were normalized against area measured. Average FcγRIIb signal for microglia and neurons were calculated based on colocalization of signal with Iba‐1‐ and NeuN‐positive structures, respectively. Unless otherwise stated, all graphs and statistical analyses were generated using GraphPad Prism (v. 8.4.3).

### 2.9 Statistics

In field electrophysiological recordings, the strength of the synaptic responses was measured as the slope of fEPSP (millivolts per millisecond). All data are represented as mean ± SEM. To test for statistical significance within group, the Wilcoxon signed‐rank test (represented as Wilcox) was applied to compare the mean normalized fEPSPs at specified time points with the fEPSP at −15 min (taken as the baseline fEPSP). To compare between different groups, the Mann–Whitney U test (represented as U test) was used. Differences were considered as statistically significant when *p* < 0.05. Nonparametric tests were selected because a Gaussian normal distribution could not always be assumed due to the small sample size per series and analyses of prolonged recordings (Pang et al., 2019; Sajikumar & Korte, 2011). “n” represents the number of slices in in vitro electrophysiology or number of animals in behavioural experiments. Slices from a minimum of 3–4 biological replicates were used for all in vitro and biochemistry experiments. Statistical comparisons for behavioural tagging were performed using unpaired Student's *t* test or one‐way ANOVA (parametric) (Krishna‐K et al., 2020; Wong et al., 2019). *p* < 0.05 was considered as the cut‐off for statistically significant differences.

## 3 RESULTS

### 3.1 Faster decay of STET‐induced L‐LTP in female APP/PS1 mice

In order to study long‐term potentiation (LTP) in APP/PS1 mice, we applied STET in four groups of animals: WT males and females, APP/PS1 males and females. In both male and female WT animals, STET application in synaptic input S1 resulted in L‐LTP that lasted for 240 min (Figure 1b,c; Figure 1b, n = 6, 1‐min Wilcox, *p* = 0.03, 1‐min U test, *p* = 0.002, 240‐min Wilcox, *p* = 0.03, 240‐min U test, *p* = 0.002; Figure 1c; n = 7, 1‐min Wilcox, *p* = 0.02, 1‐min U test, *p* = 0.0006, 240‐min Wilcox, *p* = 0.02, 240‐min U test, *p* = 0.005). We did not find a statistically significant difference in the potentiation between WT males and females at any compared time points *(*U test, *p* > 0.05; Figure 1b,c). However, in both male and female APP/PS1 mice, the same experimental design yielded a decaying form of LTP with an observable faster decay of potentiation in females compared with males (Figure 1d,e). STET resulted in an E‐LTP that lasted for 180 min in AD males (Figure 1d, n = 7, Wilcox, *p* = 0.07, U test, *p* = 0.08; 1‐min Wilcox, *p* = 0.008, 1‐min U test, *p* = 0.0002, 240‐min Wilcox, *p* = 0.25, 240‐min U test, *p* = 0.279). However, LTP in AD females resulted in an E‐LTP that lasted only 95 min (Figure 1e, n = 7, Wilcox, *p* = 0.109, U test, *p* = 0.07; 1‐min Wilcox, *p* = 0.008, 1‐min U test, *p* = 0.0002, 240‐min Wilcox, *p* = 0.469, 240‐min U test, *p* = 0.13). The faster decay of LTP in females was significant from 30 min (Figure 1d,e; 30‐min U test, *p* = 0.02). Control baseline stimulation of input S2 in all groups revealed stable potentials throughout the entire recording time period of 240 min. Overall, our results showed faster decay of STET‐induced L‐LTP in APP/PS1 females compared with males.

### 3.2 Faster decay of TBS‐induced L‐LTP in female APP/PS1 mice

**FIGURE 4:**
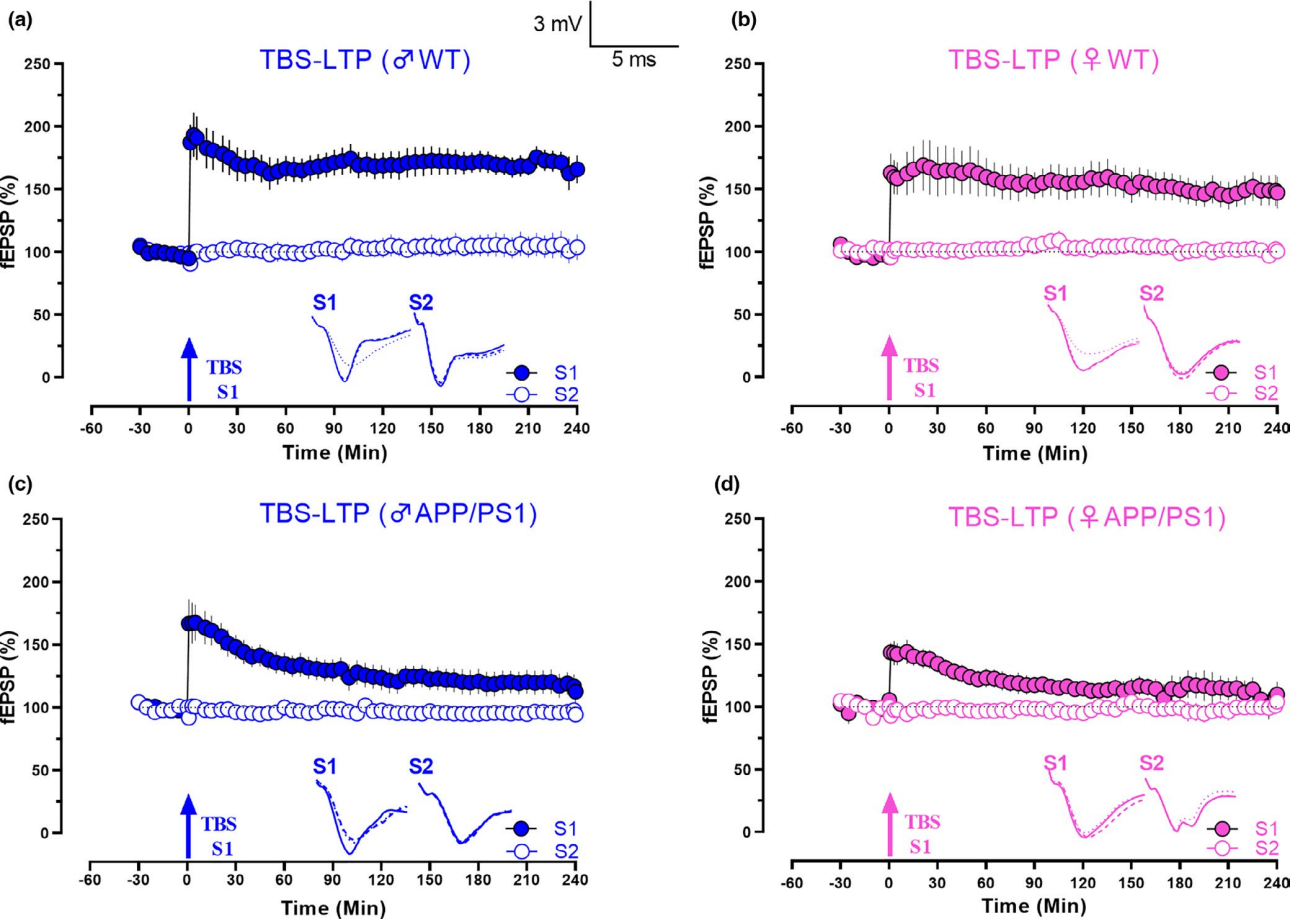
APP/PS1 females show a faster decay in TBS‐LTP. (a) Theta burst stimulation resulted in an increase in synaptic response in S1 (blue, filled circles) that lasted for 4 h, while the control input S2 (blue, open circles) remained stable for 4 h in male WT mice (n = 7). (b) Late‐ LTP was maintained for 4 h, when TBS was applied to S1 (pink filled circles) in female WT mice (n = 7). (c) TBS applied to S1 (blue, filled circles) resulted only in an E‐LTP that gradually decayed to baseline in male APP/PS1 mice (90‐min Wilcox, *p* = 0.079, U test, *p* = 0.05; n = 7). (d) TBS was delivered to S1 (pink, filled circles) in female APP/PS1 mice again showed only an early form of LTP (70‐min Wilcox test, *p* = 0.084, U test, *p* = 0.1; n = 10). Control inputs in all remained stable (open circles). Solid single arrow represents the time of induction of TBS stimulation in S1. Scale bars: vertical, 2 mV; horizontal, 3 ms. Error bars indicate ±SEM. Symbols and analogue traces as in Figure 1

Next, we examined theta burst stimulation (TBS) induced L‐LTP using 50 bursts at 5 Hz. TBS resulted in a robust L‐LTP in both male and female WT animals (Figure 4a,b), which lasted for 240 min (Figure 4a, n = 7,1‐min Wilcox, *p* = 0.016, 1‐min U test, *p* = 0.0006, 240‐min Wilcox, *p* = 0.03, 240‐min U test, *p* = 0.002; Figure 4b; n = 7, 1‐min Wilcox, *p* = 0.01, 1‐min U test, *p* = 0.0006, 240‐min Wilcox, *p* = 0.015, 240‐min U test, *p* = 0.0006). We did not observe any difference in the amplitude of LTP between male and female WT animals at any compared time points (Figure 4a,b, *p* > 0.05). As expected, we saw an impairment in TBS‐LTP in both male and female APP/PS1 mice (Figure 4c,d). TBS resulted in an E‐LTP, and the potentiation was significant until 90 min in males (Wilcox, *p* = 0.079, U test, *p* = 0.05; Figure 4c; n = 7, 1‐min Wilcox, *p* = 0.016, 1‐min U test, *p* = 0.0006, 240‐min Wilcox, *p* = 0.2188, 240‐min U test, *p* = 0.2403). In females, the potentiation remained significant only until 70 min (Wilcox test, *p* = 0.084, U test, *p* = 0.1; Figure 4d, n = 10, 1‐min Wilcox, *p* = 0.002, 1‐min U test, *p* = <0.0001, 240‐min Wilcox, *p* = 0.47, 240‐min U test, *p* = 0.72). Interestingly, we also observed a difference in the induction of TBS‐LTP in both sexes of APP/PS1 mice (Figure 4c,d; 5‐min U test, *p* = 0.02). Overall, our results showed reduced induction and faster decay of TBS‐induced L‐LTP in APP/PS1 females as compared to males.

### 3.3 Faster decay of population spike timing‐dependent plasticity in female APP/PS1 mice

Spike timing‐dependent plasticity is a phenomenon where the order and precise timing of spikes determine the direction and magnitude of plasticity and is often considered as the first law of synaptic plasticity (Dan & Poo, 2006; Feldman, 2012). Compared with STET and TBS, where the stimulus is delivered to the entire presynaptic axon, the minimal nature of STDP protocols, which requires only pairing of spikes, makes it a comprehensive learning rule for synapses (Shouval et al., 2010). We have reported earlier that coincidental pre‐ and postsynaptic stimulation induces persistent potentiation in the CA1 area of rat hippocampal slices (Pang et al., 2019). Thus, we first tested pSTDP by pairing pre‐ and postsynaptic inputs at 0 ms (S0; relative timing interval between S0 and S1 stimulations Δt = 0 ms) in male and female WT mice (Figure 2b,c). Long‐lasting potentiation was observed until 240 min in both male and female WT mice (Figure 2b, n = 6, 5‐min Wilcox, *p* = 0.03, 5‐min U test, *p* = 0.002, 240‐min Wilcox, *p* = 0.03, 240‐min U test, *p* = 0.002; Figure 2c, n = 10, 5‐min Wilcox, *p* = 0.002, 5‐min U test, *p* = <0.0001, 240‐min Wilcox, *p* = 0.009, 240‐min U test, *p* = 0.007).

**FIGURE 5:**
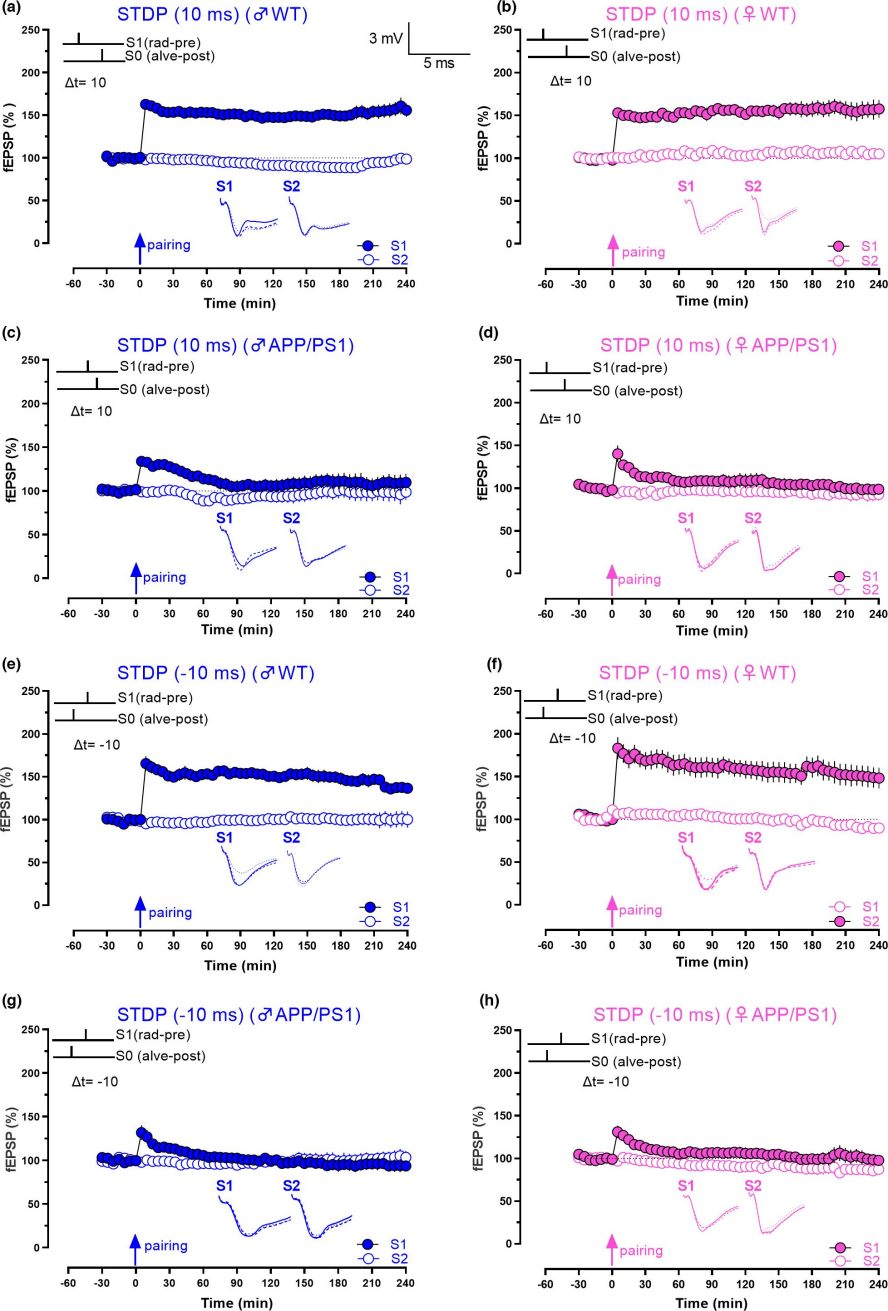
Synaptic rules for STDP are altered in APP/PS1 mice. (a) Forward pairing of pre‐ and postsynaptic stimulations at positive time interval of 10 ms led to persistent potentiation of synaptic responses in the synaptic input S1 (blue, filled circles) in male WT mice (n = 7). (b) Persistent potentiation of 4 h was observed in the synaptic input S1 (pink, filled circles) using the same experimental paradigm as in A in female WT mice (n = 7). (c) Pairing of pre‐ and postsynaptic inputs as in A and B in male APP/PS1 mice resulted only in a short‐lasting LTP in S1 (blue, filled circles; 60‐min Wilcox, *p* = 0.63, U test, *p* = 0.1; n = 6). (d) Pairing at positive time interval of 10 ms in female APP/PS1 mice resulted only in a short‐lasting potentiation in S1 (pink, filled circles; 55‐min Wilcox, *p* = 0.94, U test, *p* = 0.09; n = 6). (e) Backward pairing or repeated pairing (arrow) of alveus stimulation (S0) with subsequent Schaffer collateral stimulation in S1 (blue, filled circles) with a relative time lag of −10 ms resulted in a persistent potentiation lasting 4 h in S1 (blue, filled circles) in male WT mice. (n = 7). (f) Same experiment was repeated in female WT, which also resulted in a persistent potentiation lasting 4 h in S1 (pink, filled circles), (n = 7). (g) Backward pairing at −10 ms in male APP/PS1 mice resulted in a decremental LTP (blue, filled circles) in S1 (20‐min, Wilcox, *p* = 0.08, U test, *p* = 0.1; n = 7). (h) In female APP/PS1 mice also, only a decremental LTP was observed in S1 (pink, filled circles) after backward pairing at time interval of 10 ms (15‐min Wilcox, *p* = 0.06, U test, *p* = 0.06; n = 7). Solid single arrow represents the time of pairing. Scale bars: vertical, 2 mV; horizontal, 3 ms. Error bars indicate ±SEM. Symbols and analogue traces as in Figure 1

Next, we repeated the same experiments in APP/PS1 mice. As expected, pSTDP in both male and female mice was impaired in pSTDP (Figure 2d,e) resulting only in an E‐LTP. Statistically significant pSTDP maintained until 70 min in males (Wilcox, *p* = 0.02, U test, *p* = 0.1), and only until 40 min in females (Wilcox, *p* = 0.2, U test, *p* = 0.0830; Figure 2d; n = 6, 5‐min Wilcox, *p* = 0.03, 5‐min U test, *p* = 0.002, 240‐min Wilcox, *p* = 0.6, 240‐min U test, *p* = 0.4; Figure 2e; n = 8, 5‐min Wilcox, *p* = 0.016, 5‐min U test, *p* = 0.001, 240‐min Wilcox test, *p* = 0.2, 240‐min U test, *p* = 0.5). Significant differences in the amplitude of potentiation in pSTDP were observed between APP/PS1 males and females at 5 min and 120 min (Figures 2d,e, 5‐min U test, *p* = 0.02, 120‐min U test, *p* = 0.04), suggesting that the decay of timing‐induced plasticity at 0 ms was faster in females than in males.

### 3.4 Impaired STDP in APP/PS1 mice with forward and backward pairing of synaptic activity at 10 ms

Next, we wanted to examine whether the persistence of plasticity is affected in APP/PS1 mice by changing the timing and order of pre‐ and postsynaptic activity. Forward pairing of pre‐ and postsynaptic stimulations at a positive time interval of 10 ms (Δt = +10) in WT male and female mice resulted in persistent pSTDP that lasted 240 min (Figure 5a,b; Figure 5a, n = 7, 5‐min Wilcox, *p* = 0.0156, U test, *p* = 0.006, 240‐min Wilcox, *p* = 0.02, U test, *p* = 0.006; Figure 5b, n = 7, 5‐min Wilcox, *p* = 0.02, U test, *p* = 0.01, 240‐min Wilcox, *p* = 0.02, U test, *p* = 0.02). The potentiation observed at Δt = 10 ms in Figure 5a,b was similar to the one elicited by simultaneous pre‐ and postsynaptic stimulations as in Figure 2b,c. However, when the same set of experiments was repeated in male and female APP/PS1 mice, we observed a decremental pSTDP (E‐LTP; Figure 5c,d). In male APP/PS1 mice, the synaptic potentials remained significant until 60 min (Figure 2c, n = 6, Wilcox, *p* = 0.63, U test, *p* = 0.1), while in females, it lasted only until 55 min (Figure 5d, n = 6, Wilcox, *p* = 0.94, U test, *p* = 0.09).

Next, we explored whether backward pairing with a time interval of 10 ms has any effect on the persistence of potentiation (Δt = −10 ms). Similar to Figure 5a,b, we observed a long‐lasting pSTDP in both WT males and females that lasted 240 min (Figure 5e,f; Figure 5e, n = 7, 5‐min Wilcox, *p* = 0.03, U test, *p* = 0.002, 240‐min Wilcox, *p* = 0.04, U test, *p* = 0.04; Figure 5f, n = 7, 5‐min Wilcox, *p* = 0.04, U test, *p* = 0.002, 240‐min Wilcox, *p* = 0.03, U test, *p* = 0.002). However, when the same experiments were repeated in APP/PS1 mice, the pSTDP was impaired in both males and females resulting only in a short‐term potentiation (STP; Figure 5g,h). In APP/PS1 males, statistically significant potentiation was maintained until 20 min (Figure 5g, n = Wilcox, *p* = 0.08, U test, *p* = 0.1), while in females, potentiation sustained only until 15 min (Figure 5h, n = 6, Wilcox, *p* = 0.06, U test, *p* = 0.06).

### 3.5 Associative memory showed higher decline in females than in males

In order to study whether the synaptic plasticity deficits observed in hippocampal slices were reflected in hippocampus‐dependent memory tasks, we studied associative memory in WT and APP/PS1 mice using the behavioural tagging (BT) paradigm (Moncada & Viola, 2007; Wong et al., 2019). In BT, spatial novelty in the form of an open field (OF) induces the synthesis of plasticity‐related proteins that will be captured by a subsequent weak stimulus in the form of a mild foot shock (weak memory), thus allowing weaker memories to be consolidated into long‐term memories according to the synaptic tagging and capture hypothesis (Moncada & Viola, 2007; Shetty & Sajikumar, 2017). It has been demonstrated earlier that a weak memory can be consolidated into a strong memory, if it occurs in close association with a strong stimulus (OF) (Moncada & Viola, 2007). Memory was measured as the latency to step down onto the bars, and a longer step‐down latency indicates a stronger memory association. The time points are typically used to assess short‐term memory (STM: 1 h after the training session), long‐term memory (LTM: 24 h after training) and remote LTM (7 days after training), respectively.

In the control group (weak IA alone), four sets of animals (WT male and female and APP/PS1 male and female) were subjected to a weak foot shock and tested for inhibitory avoidance (IA) by measuring step‐down latency at various time points after training (1, 24 h, 7 days; Figure 3a). In the experimental BT group (OF before weak IA), another four sets of mice were subjected to the same conditions as the control group, except that they were first exposed to OF for 10 min prior to the application of a weak foot shock (Figure 3b).

In the control group, when latency for training was compared with latency for test sessions, significant difference was observed at 1 h, but not at 24 h and 7 days in WT and APP/PS1 males (WT males; Figure 3a, blue solid bars, 1 h, *p* = <0.0001; 24 h, *p* = 0.06; 7 days, *p* = 0.3; APP/PS1 males; Figure 3a, open blue bars, 1 h, *p* = <0.0001; 24 h, *p* = 0.09; 7 days, *p* = 0.9). Similar findings were observed in WT and APP/PS1 females (WT females; Figure 3a, solid pink bars, 1 h, *p* = <0.0001; 24 h, *p* = 0.2; 7 days, *p* = 0.7; APP/PS1 females; Figure 3a, open pink bars, 1 h, *p* = 0.003; 24 h, *p* = 0.08; 7 days, *p* = 0.6). Our results show that mild foot shock induces STM but not LTM or remote memories in all groups of mice.

Notably, while both APP/PS1 males and females recalled weak IA learning at 1 h, the mutant mice still exhibit significant impairment when compared to their WT counterparts (male WT [solid blue bars] vs male APP/PS1 [open blue bars] at 1 h, U test, *p* = 0.0023; female WT [solid pink bars] vs female APP/PS1 [open pink bars], U test, *p* = 0.0022).

In the BT group, where OF preceded weak IA, both WT males and females showed STM (1 h), LTM (24 h) and remote memory lasting 7 days (Figure 3b, solid blue bars and solid pink bars, respectively; WT males; 1 h, *p* = <0.0001; 24 h, *p* = <0.0001; 7 days, *p* = <0.0001; WT females; 1 h, *p* = <0.0001; 24 h, *p* = <0.0001; 7 days, *p* = <0.0001). The APP/PS1 males showed LTM at 24 h, but not remote memory at 7 days (APP/PS1 males; open blue bars, 1 h, *p* = 0.003; 24 h, *p* = 0.004; 7 days, *p* = 0.1), whereas in APP/PS1 females, both LTM and remote memory were abolished (APP/PS1 females; 1 h, *p* = 0.003; 24 h, *p* = 0.3; 7 days, *p* = 0.98).

APP/PS1 mice in the BT group showed a deficit in learning relative to WT mice of the same sex at all time points (Figure 3b, WT male [solid blue bars] vs APP/PS1 males [open blue bars] at 1 h, U test, *p* = 0.001; WT female [solid pink bars] vs APP/PS1 females at 1 h, U test, *p* = 0.0007). When latency was compared at 24 h and 7 days post‐training, similar results were observed (24 h, WT male vs APP/PS1 males, U test, *p* = 0.0003, WT female vs APP/PS1 female, U test, *p* = 0.0007); 7 days post‐training, WT male vs APP/PS1 males, U test, *p* = 0.0003, WT female vs APP/PS1 female, U test, *p* = 0.0007).

In addition, the BT paradigm also uncovered subtle sex‐specific differences at 24 h and 7 days in APP/PS1 mice, when compared between the groups (APP/PS1 males vs APP/PS1 females; 24 h, U test, *p* = 0.009; 7 days, U test, *p* = 0.007; in the control group, male APP/PS1 [open blue bars] vs female APP/PS1 [open pink bars] at 1 h, U test, *p* = 0.0076; Figure 3a). Comparison of step‐down latency between WT males and females did not show significant difference at any points.

Our results indicate that exposing mice to OF prior to weak IA stimuli enhanced learning across all time points for both WT males and females. However, in APP/PS1 males, STM and LTM remained intact while remote memory was abolished. In contrast, female APP/PS1 mice showed STM but LTM and remote memory were abolished. Thus, female APP/PS1 are able to acquire IA memory, while they show impairment in the formation of IA‐LTM.

### 3.6 Activation of neuroinflammatory genes and downregulation of plasticity‐related genes in APP/PS1 female mice

**FIGURE 6:**
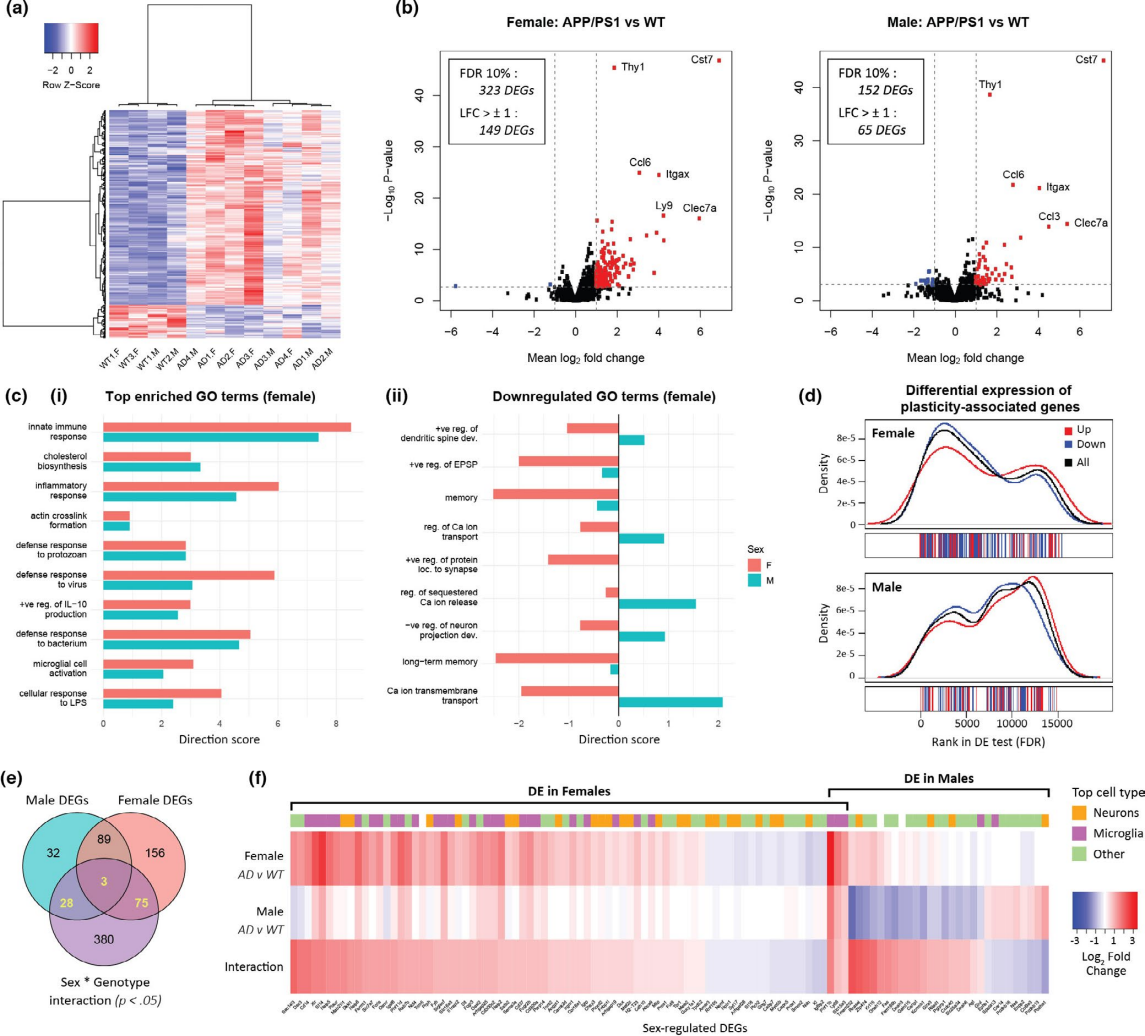
APP/PS1 female hippocampus is enriched in neuroinflammatory genes and depleted in genes associated with neuronal plasticity and memory. (a) Heatmap showing differentially expressed genes (DEGs) between APP/PS1 (AD) and wild‐type (WT) mice (n = 445; FDR <0.1). (b) Volcano plots showing log2 fold changes (LFCs) compared with *p*‐values from AD vs WT differential expression tests for female and male mice. (c) Enriched Gene Ontology terms sorted by *p*‐value in females (most significantly enriched at the top). Direction scores (DS) indicate overall up‐ or downregulation of genes in each term (abbreviations: +ve, positive; ‐ve, negative; reg., regulation; LPS, lipopolysaccharide; dev., development; EPSP, excitatory postsynaptic potential; Ca, calcium; loc., localization). (ci) Top enriched terms in females. (cii) Selected downregulated enriched terms associated with memory and plasticity (9/15 top terms with DS<0; *p* < 0.01). (d) Genes associated with the GO term “regulation of synaptic plasticity” ranked by FDR in DE tests comparing genotypes for each sex. Genes with higher expression in AD mice are indicated in red, and lower in blue. Density plots represent the probability that plasticity genes are found at each point in the ranked list. (e) Overlap between DEGs in male and female AD vs WT mice (FDR <0.1) and genes with a potential interaction effect (*p* < 0.05), indicating significantly different effects of genotype between sexes. The N = 106 genes overlapping between groups are sex‐regulated DEGs. (f) Heatmap of the sex‐regulated DEGs identified in 6E. Plot shows LFCs for females (AD v WT), males (AD v WT) and the interaction effect. A positive interaction effect indicates that a gene is more highly expressed in female than in male AD mice relative to WT mice of same sex, that is, upregulated in females or downregulated in males. For each gene, the cell type (neuron, microglia, or other) with highest expression in the transcriptome database of (Zhang et al., 2014) is indicated above the plot

We investigated gene expression in the hippocampus of 4‐ to 5‐month‐old APP/PS1 and WT subjects of both sexes using RNAseq. We detected 323 and 152 differentially expressed genes (DEGs) in female and male APP/PS1 mice, respectively, as compared to WT (with an overlap of 92 DEGs; FDR <0.1), indicating that there is a greater effect of AD pathology on gene expression in female mice at this age (Figure 6a,b and Figure S1a). For both sexes, the top upregulated genes are similar and associated with the immune system: *Thy1*, *Ccl6*, *Itgax*, *Clec7a and Cst7*.

To investigate biological processes that may be affected by the differential transcriptional response between male and female mice, we performed gene ontology (GO) analysis using Kolmogorov–Smirnov tests on genes ranked by FDR. As this method does not require a DE threshold, results can be directly compared between sexes. We found that the top enriched terms in female mice indicated robust activation of the immune system, including increased cytokine production, and activation of microglia and innate inflammatory responses (Figure 6ci). The upregulated inflammatory genes include *Cst7*, *Tyrobp*, *Trem2*, *Tlr2*, *Ctss and* members of the *Naip* family. Similar to female mice, immune genes were also significantly upregulated in male mice (Figure 6ci) although the top enriched terms in male mice were also linked to a range of other processes including G protein‐coupled receptor signalling and cellular adhesion (Figure S1b). Another GO term strongly enriched in both sexes is related to cholesterol biosynthesis, a process that has been reported to be widely dysregulated in AD (Sun et al., 2015).

While the majority of GO terms described upregulated sets of genes, it is notable that the downregulated gene sets (direction score [DS] <0) in female mice were primarily associated with memory and plasticity, including regulation of calcium release and transport, and regulation of the development of dendritic spines and neuronal projections (Figure 6cii). These gene sets were not downregulated in male APP/PS1 mice (Figure 6cii). To extend this analysis, we examined the differential expression of all genes annotated to the GO term “regulation of synaptic plasticity” (n = 208) and found that these genes were both significantly altered and predominantly downregulated in females (*P* = 1.3 × 10^−5^, DS = −4.02), but not in males (*p* = 0.016, DS = −0.14; Figure 6d). In summary, while immune genes are upregulated in both sexes, plasticity‐related genes are mainly downregulated in female APP/PS1 mice.

We next identified genes that were affected differently by APP/PS1 pathology in male and female mice. Of the protein‐coding genes that were differentially expressed between genotypes in either or both sexes, 106 had an interaction effect with a *P*‐value <0.05 (Figure 6e). Approximately three quarters of these sex‐regulated DEGs were differentially expressed only in females (n = 75) with most of them upregulated (n = 58; Figure 6e,f). Once again, consistent with our overall observations, many of these female‐specific DEGs are highly expressed in microglia and involved in immune and inflammatory processes (Figure 6f). Notably, some of the female‐specific DEGs are expressed in neurons and implicated in AD, including genes known to regulate synaptic plasticity; in particular, *Mef2c* (transcription factor), *Sema3a* (axon guidance), *FcγRIIb* (Ig receptor) and genes reportedly involved in AMPAR trafficking, *Syt17*, *Nptx1* and *Myo5b* (Figure S1c) (Figueiro‐Silva et al., 2015; Kam et al., 2013; Ruhl et al., 2019; Sao et al., 2018; Tansey et al., 2018; Wang et al., 2008).

### 3.7 APP/PS1 female mice have more Aβ plaques and Iba‐1‐positive microglia in the hippocampus

**FIGURE 7:**
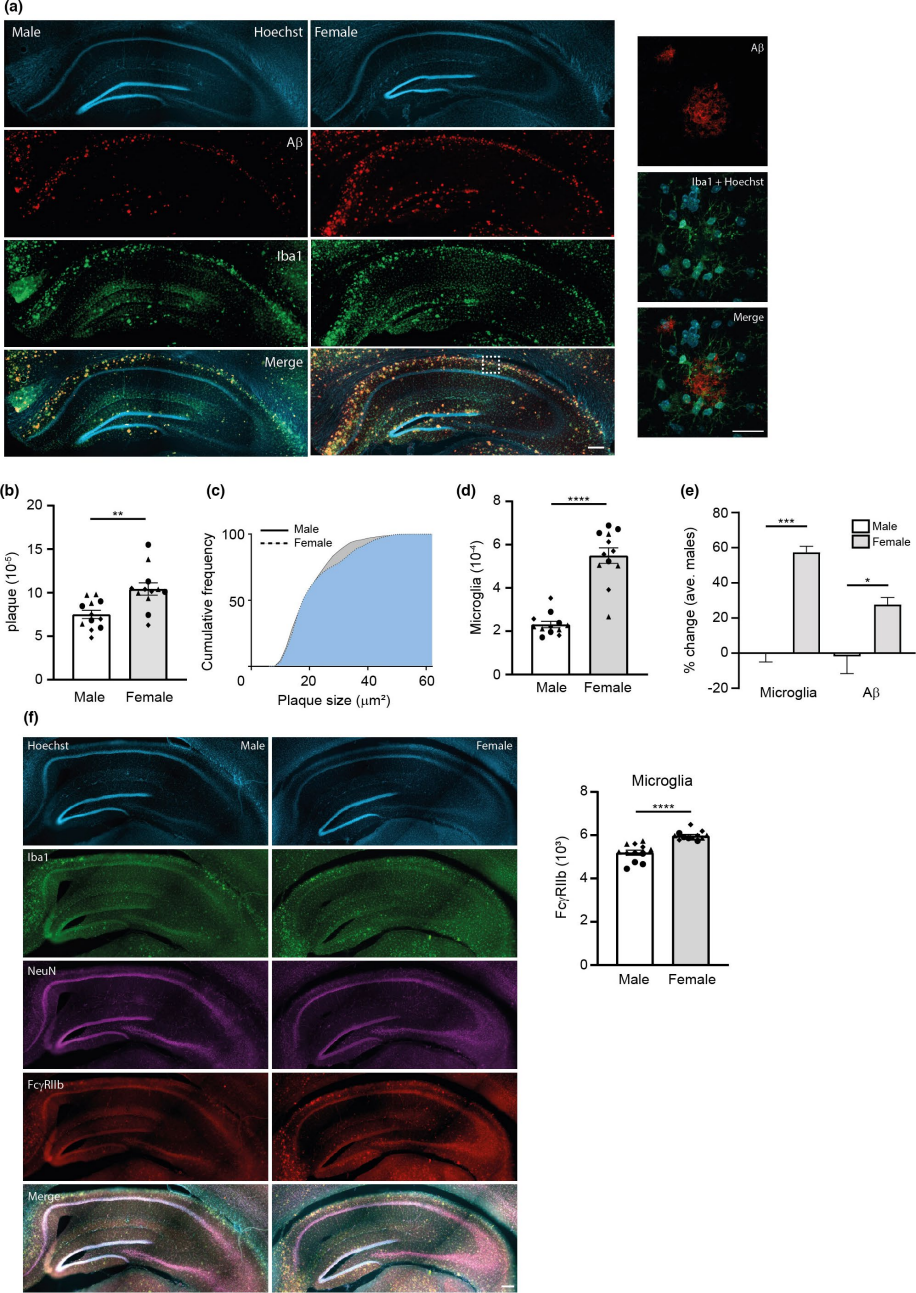
APP/PS1 female mice have more Aβ plaques and microglia. (a) Hippocampal slices from APP/PS1 mice from both sexes were processed and immunolabelled with antibodies against Iba‐1 (green), Aβ (red) and Hoechst nuclear dye (cyan; scale bar: 200 µm). The white box shows a magnified region of the CA1 cell body layer (right most panels; scale bar: 20 µm). For analysing immunolabelled structures, a total of four non‐overlapping hippocampal coronal sections per animal were quantified (labelled as different shapes), three animals per group were imaged with a slide scanner and quantified. Graphs show Aβ plaque burden (b) and total microglia counts (d) normalized over area of measurement, as well as cumulative frequency of plaques sizes measured across all slices (males, n = 6060; females, n = 7163); (c). The per cent change in plaque burden and microglia between male and female mice for each animal (n = 3) was also quantified and graphed (e). (f) Hippocampal slices were immunolabelled with antibodies against Iba‐1 (green), NeuN (purple), FcγRIIb (red) and nuclear dye, Hoechst (cyan). The Iba‐1‐positive FcγRIIb signals were quantified for each slice and plotted on the bar graph (scale bar: 200 µm). Statistical analyses of all group data were performed using unpaired Student's *t* test with Welch's correction (****p<0.0001, ***p<0.001 and **p<0.01)

Our transcriptome analysis indicates a robust upregulation of neuroinflammatory and microglial genes that could serve as a molecular basis for the greater impairment in long‐term plasticity in female AD mice. To verify that the immune system is differentially activated in male and female mice, we sectioned the hippocampus of AD mice from both sexes (4–5 months) and performed immunohistochemistry using antibodies targeted against Aβ (MOAB‐2) and microglia (Iba‐1). Quantification of Aβ plaques and Iba‐1‐positive microglia indicated that female APP/PS1 mice have an accelerated Aβ pathology with an increase not only in plaque burden and plaque size but also in Iba‐1‐positive microglia in the hippocampus (Figure 7a‐e).

FcγRIIb encodes an Fc receptor that is upregulated in activated microglia in response to brain injury (Quan et al., 2009). In our transcriptome profile, FcγRIIb expression showed sex‐specific differences via mRNAseq and we immunostained for FcγRIIb protein in hippocampal slices to examine the expression pattern of the protein in neurons and microglia. Our imaging results detected a significant increase in FcγRIIb protein expression in female APP/PS1 mice, specifically in the microglial population, but not in neurons (Figure 7f and Figure S1d). We conclude that the sex‐specific difference in FcγRIIb expression identified via mRNAseq is attributed primarily to an increase in AD pathology driven by activation of microglia.

## 4 DISCUSSION

In AD, females are disproportionately affected in terms of enhanced pathology, more severe cognitive decline and other clinical manifestations during the progression of the disease (Arnsten et al., 2021; Koran et al., 2017; Ward et al., 2012). In agreement with this observation, our results in an animal model of AD showed that both activity and time‐dependent plasticity decline faster in females as compared to males. Our behavioural assay using the BT paradigm showed that overall, APP/PS1 mice have strong deficits in memory formation at all time points tested. Additionally, the assay revealed that female APP/PS1 mice have slightly more robust deficits in LTM and formation of associative memories as compared to the male APP/PS1 mice. Since LTP remains a prominent cellular model for the persistence of long‐term memories, a more rapid decay of LTP in female APP/PS1 mice may be associated with a more severe cognitive impairment or memory loss in female AD patients (Barnes et al., 2005; Cohen et al., 1993; Ott et al., 1996). In trying to elucidate the molecular basis for the sex‐specific differences in long‐term plasticity, our transcriptome analysis revealed that APP/PS1 mice exhibit a strong neuroinflammatory response and enhanced microglial activation along with an increase in Aβ pathology. We also found that plasticity genes were downregulated in female compared with male APP/PS1 mice, suggesting that the accelerated Aβ pathology in females likely disrupts the expression of plasticity‐related proteins (PRPs), leading to the accelerated decay of LTP and memory in females compared with males. The downregulation of PRPs could be due to alterations in the function of NMDA and AMPA receptors, as sex differences are found in synaptic glutamate signalling (Mota et al., 2014; Qu et al., 2020; Wickens et al., 2018). An increase in glutamate levels severely affects AD males compared with females as they exhibit lower levels of GluA2‐containing AMPA receptor subunits (Wickens et al., 2018). Thus, our findings support the observation that females are more vulnerable to synaptic and memory dysfunctions during progression of the disease.

Sex differences in neuronal plasticity have been described. Rodents are reported to have sex differences in spine density across different brain regions (Forlano & Woolley, 2010; Gould et al., 1990; Woolley et al., 1990), and the composition of the synaptic proteome varies between male and female mice (Distler et al., 2020). Many of the sex differences in structural plasticity are driven by hormones, and hence, their influence is more pronounced in females (Hyer et al., 2018). For example, oestrogen increases spine density in an extracellular signal‐regulated protein kinase (ERK)‐dependent manner and regulates gene transcription and protein synthesis (Nilsson et al., 2001). Ovariectomized female rodents also displayed a decline in spine density that is reversed by hormonal supplements (Gould et al., 1990; MacLusky et al., 2005). LTP in the Schaffer collateral pathway is also modulated by oestrogen and expression of endogenous estrogen receptor α (ERα) in females, but not in males. Infusion of oestradiol facilitated LTP and synaptic signalling by ERα and ERβ in females, but not in males (Wang et al., 2018). Moreover, activation of kinases such as Src, ERK and TrkB is modulated by expression of ERα in females (Wang et al., 2018). Given the extensive influence of hormones on neural plasticity, any hormone imbalance or disruption in hormone‐responsive plasticity mechanisms in AD conditions could explain the faster decay of LTP and decline in associative memories in AD females (Lu et al., 2019). While sex hormones decline during normal ageing, in AD, there is evidence that brain levels of sex hormones (Rosario et al., 2004, 2011; Yue et al., 2005) along with sex hormone receptor expression, function and subcellular distribution are further compromised (Long et al., 2012; Lu et al., 2003). While the relationship between hormones and its impact on AD remains to be clarified, altered brain hormone levels and its decline in responsiveness in AD may provide clues in understanding sex‐specific differences in impaired neural plasticity and memory loss.

It should be noted that the oestrous cycle of female animals did not impact our LTP recordings, as we did not find any significant differences in potentiation and decay across the various oestrous cycles (Figure S2). The involvement of the oestrous cycle towards synaptic plasticity and LTP remains unresolved with some studies reporting enhancement of CA1 LTP during specific pro‐oestrous periods while others showing no significant differences in CA1 LTP (Hong et al., 2016; Warren et al., 1995).

Our transcriptome profiles of the male and female APP/PS1 hippocampus indicated an overall increase in gene expression associated with the immune response. Many of the canonical markers for microglial activation and neuroinflammation were upregulated in both sexes, while female mice displayed a twofold increase in differentially expressed genes compared with their male counterparts, suggesting a strong sex‐specific difference in response to AD pathology. While slice recordings indicated that both males and females have impaired long‐term plasticity, it was surprising to see that only female mice showed a broad downregulation of genes associated with plasticity and memory. Collectively, these changes in gene expression may account for the accelerated decay of late LTP and STDP. It should be noted that while the expression of plasticity gene sets was overall reduced in APP/PS1 females, we detected relatively few changes in individual genes. This could reflect a gradual dysregulation of diverse molecular processes associated with neuronal plasticity rather than single‐gene disruptions in APP/PS1 female mice. In addition, we also identified a set of female‐specific, differentially expressed neuronal genes previously linked to AD pathology including *Sema3a* and *Mef2c* (Abad et al., 2006; Liang et al., 2010). Both genes are known regulators of neuronal plasticity. *Sema3a* not only encodes a secreted protein that is critical for axon guidance but also regulates dendrite branching and spine maturation (Morita et al., 2006). Mef2c is a well‐known activity‐dependent transcription factor involved in learning and memory (Barbosa et al., 2008). In addition, three of the female‐specific DEGs that had reduced expression in APP/PS1 mice, including Syt17, are involved in AMPAR trafficking during synaptic plasticity, suggesting this function may be disrupted. Intriguingly, expression of both Mef2c and Syt17 has been shown to be affected by androgens in neurodevelopment, suggesting that the effect of sex hormones on plasticity gene expression may be a key factor predisposing females to AD (Lombardo et al., 2020). Future studies targeting differentially expressed genes in female mouse models for AD that are independent of the immune system but associated with neuronal plasticity might prove to be informative.

The brain slice immunostaining validates our gene expression data, which points to females showing advanced Aβ pathology accompanied by a stronger inflammatory response. This accelerated progression has been reported in other mouse models and in AD patients (Wang et al., 2020; Yang et al., 2018). As previously discussed, sex differences in synaptic plasticity may be responsible for synaptic dysfunction in AD, but the accelerated pathology and the increased activation of microglia can also contribute towards impairments in neural plasticity. Microglia are essential for synaptic pruning, and aberrant microglial‐mediated engulfment of synapses in response to elevated Aβ plaque levels could conceivably impact synaptic transmission (Hong et al., 2016; Parihar & Brewer, 2010; Raghuraman et al., 2019). Alternatively, elevated Aβ levels in the extracellular microenvironment can also directly impact synaptic efficacy. For example, Aβ plaques are known to disrupt the synthesis of plasticity‐related proteins required for the maintenance of LTP (Sharma et al., 2017). Thus, a higher Aβ plaque burden might account for the lower expression of plasticity proteins. Interestingly, FcγRIIb expression has been reported not only in microglia but also in circulating macrophages and other myeloid populations, raising the possibility that the increased expression in female AD mice could also be attributed to the infiltration of circulating macrophages (Quan et al., 2009).

Earlier studies have shown that soluble Aβ42 oligomers significantly blocked the induction and maintenance of HFS‐LTP, but not TBS‐LTP, thus showing that sex difference depends on the stimulation protocol (Smith et al., 2009). Sex difference in the amplitude of LTP also depends on hippocampal projections, as a robust sex difference in the magnitude of LTP was observed in DG after perforant path stimulation (Maren, 1995). Similar findings were also observed from timing‐induced plasticity, as pSTDP was impaired in both male and female hippocampal CA1 regions with female APP/PS1 mice showing a more prominent decay. Similarly, a study in human AD patients failed to induce STDP in the cortico‐cortical connections (Di Lorenzo et al., 2018). Also consistent with our findings, Garad and colleagues reported that STDP in APP/PS1 mice was impaired in an Aβ plaque distance‐dependent manner (Garad et al., 2021), suggesting that a higher Aβ load in female APP/PS1 mice might be a reason for the faster decay we observed in slice physiology and in the BT paradigm.

In agreement with our LTP and STDP findings, our behavioural experiments also revealed that APP/PS1 female mice are able to acquire IA memory, while they show impairment in the formation of IA‐LTM results. Previous studies have already shown that APP/PS1 mice have long‐term spatial memory deficits (Krishna‐K et al., 2020), but in order to uncover sex‐specific impairment in associative memories, we employed the BT paradigm, that is known to capture subtle behavioural differences that are normally masked in standard memory tasks (Gros & Wang, 2018). We proposed earlier that the deficit in associative memory in APP/PS1 mice may occur due to altered expression of plasticity‐related proteins (PRPs) or impaired setting of synaptic tags in neurodegenerative neural networks. We found that plasticity‐related genes were downregulated in females compared with male APP/PS1 mice. Moreover, the “tag” setting processes seemed not to be impaired because STM was intact in all groups. However, we cannot rule out the possibility that STM can be sustained by other molecular/cellular processes that do not include tag setting. In line with that, Gros and Wang reported that novelty did not facilitate long‐term memory persistence in middle‐aged rats [i.e., behavioural tagging was impaired] and they concluded that this deficit was most likely due to impairments in tag setting rather than PRP synthesis in early ageing (Gros & Wang, 2018). In our studies, we observed that APP/PS1 mice were able to acquire memories, revealing the ability to set learning tag, while impairment of associative memory suggests a reduction in the availability of PRPs. Nevertheless, APP/PS1 mice had significantly shorter latencies at all key time points, indicating that tag setting process may be impaired partially. Our results show enhanced microglial activation in AD mice, females in particular are strengthened by our earlier finding showing the specific role of microglia in tag setting and STC (Raghuraman et al., 2019). We also observe a faster decay in LTP in APP/PS1 female mice, which suggests the possibility that the downstream mechanism of tag–PRPs interactions may have sex‐specific differences that warrant further investigations. It should be noted that we used the same group of animals for testing memory at different time points (1, 24 h and 7 days), which may trigger extinction memory (de Carvalho Myskiw et al., 2013). However, we did not observe any changes in memory retention in WT males and females in the BT paradigm after 24 h and 7 days postfoot shock, allowing us to rule out extinction. This finding is consistent with our previous reports (Krishna‐K et al., 2020; Wong et al., 2019).

In conclusion, our results showed that a stronger inflammatory response coupled with downregulated expression of plasticity factors in the hippocampus of AD females might underlie synaptic plasticity deficits that result in faster memory decline in AD females compared with males. As synaptic dysfunction is an early event of AD, strategies to detect early decay of plasticity, memory and associated molecular signatures may help detect the onset of neurodegenerative diseases such as AD.

## CONFLICT OF INTEREST

The authors declare that they have no conflict of interest.

## AUTHOR CONTRIBUTIONS

SN, JRG, THC and SS conceived and coordinated the study. SN, JRG, THC and SS wrote the paper. SN, MVP and RPA performed and analysed the electrophysiology experiments. JRG and VB performed and analysed the transcriptome and immunohistochemistry experiments. SN performed and analysed the behavioural experiments. YSC performed dissections for immunohistochemistry experiments.

## Supporting information

Fig S1

Fig S2

Supplementary Material

Supplementary Material

## Data Availability

All RNAseq datasets can be uploaded from NCBI GEO: GSE186710.
