# Supplementary figures and images for: Sex‐specific accelerated decay in time/activity‐dependent plasticity and associative memory in an animal model of Alzheimer's disease
Source: Aging Cell. 2021 Nov 18;20(12):e13502. doi: 10.1111/acel.13502 (PMC8672784; doi:10.1111/acel.13502)

S. Fig. 1

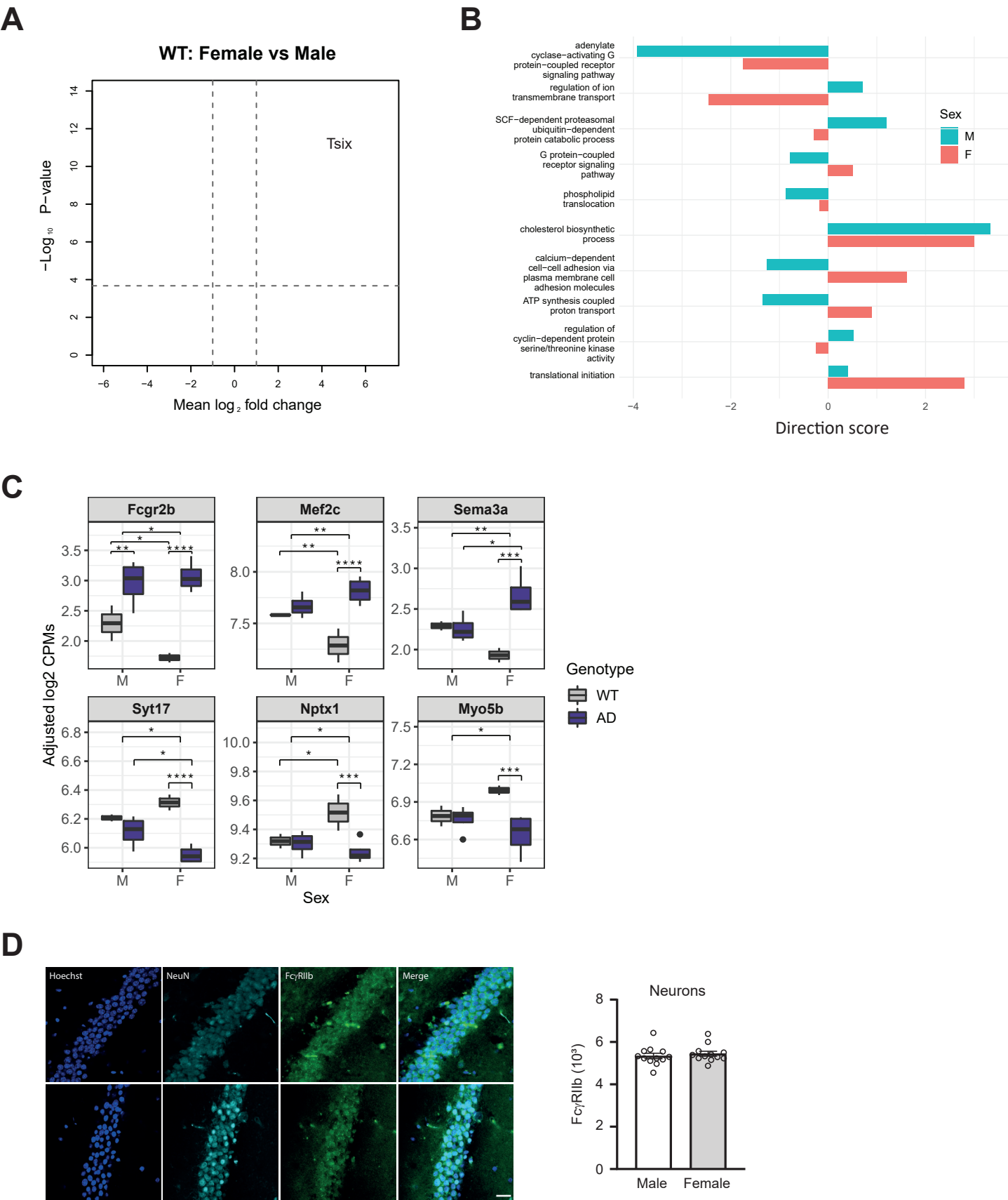

Supplement: Supplementary file 1 — Fig S1 [file ACEL-20-e13502-s002.pdf]

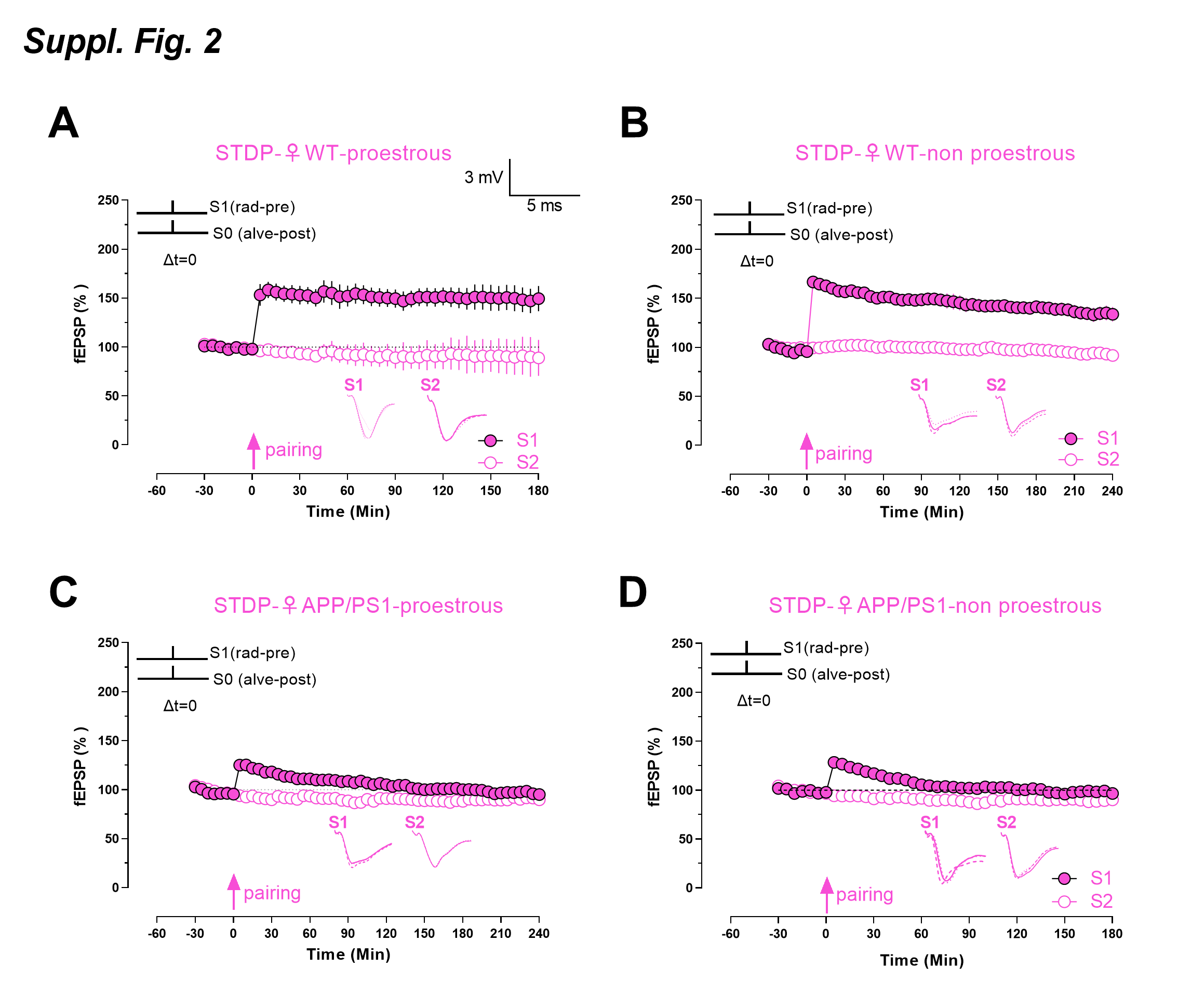

Supplement: Supplementary file 2 — Fig S2 [file ACEL-20-e13502-s001.tif]
