# Supplementary material for: Sex‐specific accelerated decay in time/activity‐dependent plasticity and associative memory in an animal model of Alzheimer's disease
Source: Aging Cell. 2021 Nov 18;20(12):e13502. doi: 10.1111/acel.13502 (PMC8672784; doi:10.1111/acel.13502)
Supplement: Supplementary file 3 — Supplementary Material [file ACEL-20-e13502-s003.pdf]

Supplementary Figure Information

LTP does not vary across different stages of estrous cycles

Hippocampal slices were classified as high estrogen (proestrous) or low estrogen (estrous, metestrous and diestrous) and pSTDP were compared between the two groups in both WT and APP/PS1 female mice (Suppl. Fig.2). pSTDP was induced by simultaneous stimulation of S1 and S0 as in Fig. 3A. Statistically significant potentiation was observed immediately after pairing at Δt = 0 ms in S1of WT females in both proestrous and non proestrous periods ($P<0.05$) (Suppl. Fig. 2A and 2B). The potentiation remained statistically significant until 240 min in S1 (solid pink circles) while the control input S2 (open pink circles) remained stable throughout the recording. We compared the potentiation between proestrous and non proestrous periods (Suppl. Fig 2A and 2B) and we did not find any significant difference between the two at all-time points (U-test,5 min, *P*=0.54, 30 min *P*=0.93, 60 min, *P*=>0.99, 120 min *P*=0.66, 180 min *P*=0.18, 240 min *P*=0.0.17). We studied the pSTDP at 0 ms in APP/PS1 females at proestrous (Suppl. Fig. 2C) and non proestrous periods (Suppl. Fig. 2D). It resulted in impairment of LTP in both proestrous and non proestrous, while we did not observe any significant differences in potentiation and decay between the proestrous and non proestrous cycles of APP/PS1 mice (Suppl. Fig. 2C and 2D) (U-test, 5 min, *P*=0.13, 30 min *P*=0.54, 60 min, *P*=0.93, 120 min *P*=0.90, 180 min *P*=0.81, 240 min *P*=0.0.81).
