# Supplementary material for: Sex‐specific accelerated decay in time/activity‐dependent plasticity and associative memory in an animal model of Alzheimer's disease
Source: Aging Cell. 2021 Nov 18;20(12):e13502. doi: 10.1111/acel.13502 (PMC8672784; doi:10.1111/acel.13502)
Supplement: Supplementary file 4 — Supplementary Material [file ACEL-20-e13502-s004.docx]

**Supplementary Figure Legends**

**Suppl. Fig. 1**

**(A)** Volcano plot of sex differences in wild-type mice, showing log2 fold changes (LFCs) compared to p-values for differential expression, excluding genes with LFC> ±8 (5 Y-chromosome genes and Xist).

**(B)** Top Gene Ontology terms enriched in males sorted by p-value (most enriched at the top). Direction scores indicate overall up- or downregulation of genes in each term in male and female APP/PS1 vs wild-type mice.

**(C)** Boxplots showing batch-corrected log_2_ counts per million (CPMs) in each condition for sex-regulated differentially expressed genes of interest: *Fcgr2b, Mef2c*, *Sema3a, Syt17, Nptx1, and Myo5b*. Differential expression tests were conducted between genotypes for each sex, between sexes for each genotype, and for the interaction effect [*(AD.F - WT.F) - (AD.M - WT.M*)] (**** p<0.0001; *** p<0.001; ** p<0.01, *p<0.05).

**(D)** Hippocampal APP/PS1 slices were immunolabeled with antibodies against NeuN (cyan) and FcγRIIb (green) and stained with Hoechst (blue). The NeuN positive FcγRIIb signal in the CA1 region was quantified for each slice and plotted on the bar graph (Scale bar: 20 µm).

**Suppl. Fig. 2**

(A) pSTDP by pairing pre and post synaptic inputs at 0 ms (S0; relative timing interval between S0 and S1 stimulations Δt = 0 ms) in female WT at proestrous. pSTDP in proestrous female WT resulted in a persistent input specific long-lasting LTP that lasted for 4 h in S1 (pink filled circles) and the unpaired input S2 (pink, open circles) remained stable (n=6). (B) pSTDP at Δt= 0 ms in non proestrous female WT also resulted in long-lasting potentiation (n=6). (C) pSTDP in APP/PS1 mice during the proestrous cycle resulted in a decremental LTP (n=7). (D) While in APP/PS1 mice at non proestrous cycle pairing at 0 ms also resulted an E-LTP (n=7). Control input S2 remained stable in all experiments (open pink bars). Scale bars: vertical, 2 mV; horizontal, 3 ms. Error bars indicate ±SEM. Symbols and analog traces as in Figure 1.
